# The Causal Role of Adiposity in Mental Illness: A Systematic Review and Meta‐Analysis of Mendelian Randomization Studies

**DOI:** 10.1111/obr.70156

**Published:** 2026-05-19

**Authors:** Min Gao, Paul J. Doody, Dimitrios A. Koutoukidis, Yebo Yu, Paul Aveyard, Richard Stevens, Padraig C. Dixon

**Affiliations:** ^1^ Nuffield Department of Primary Care Health Sciences University of Oxford Oxford UK; ^2^ National Institute for Health Research Applied Research Collaboration Oxford and Thames Valley Oxford University Hospitals, NHS Foundation Trust Oxford UK; ^3^ National Institute for Health Research Oxford Health Biomedical Research Centre Warneford Hospital Oxford UK; ^4^ Discipline of Public Health and Primary Care, Institute of Population Health, School of Medicine Trinity College Dublin, the University of Dublin Dublin Ireland; ^5^ National Institute for Health Research Oxford Biomedical Research Centre Oxford University Hospitals, NHS Foundation Trust Oxford UK; ^6^ School of Public Health Peking University Health Science Centre Beijing China

**Keywords:** adiposity, causality, Mendelian randomization, mental illness

## Abstract

**Objective:**

This study aimed to examine the causal effect of adiposity on mental illnesses by summarizing and assessing evidence from Mendelian randomization (MR) studies.

**Method:**

Searches were conducted on Embase, Medline, and Web of Science from database inception to September 22nd, 2025. Studies using MR study designs that estimated adiposity measures including body mass index, abdominal adiposity, peripheral adiposity, or body composition in relation to mental illnesses were included. Outcomes were the presence or severity of depression, anxiety, eating disorders, bipolar disorder, obsessive compulsive disorder, post‐traumatic stress disorder, schizophrenia, and related psychotic disorders. Study quality was assessed with a scoring system reflecting MR study guidelines. Data were pooled in meta‐analyses using random‐effects models. Subgroup analyses were conducted by sex.

**Results:**

Fifty‐seven studies with 215 MR estimates were included in the systematic review, and 23 studies with 44 MR estimates were included in meta‐analyses. Pooled estimates suggested that general adiposity was causally associated with depression (OR: 1.09, 95% CI, 1.02–1.15, *p* < 0.001; *I* ^2^ = 94%), but the effect size was modest and there was high heterogeneity. Subgroup differences by sex in the causal relationship were not observed (*p* = 0.318). There was suggestive evidence for causal associations of adiposity with schizophrenia and OCD; however, these analyses were characterized by high imprecision and heterogeneity. The evidence for other mental illnesses remains unclear.

**Conclusion and Relevance:**

General adiposity appears to be causally associated with depression, suggesting psychological benefits of weight management. Evidence for causal associations between adiposity and other mental illnesses remains suggestive or uncertain.

## 1 Introduction

One in eight people live with a mental illness worldwide, accounting for nearly one billion people [1]. The association between adiposity and mental illness is well established. Approximately 20%–60% of people with obesity experience at least one form of mental illness [2]. Adipose tissue is an endocrine organ that releases cytokines and other molecules that influence immune activation, insulin resistance, and lipid metabolism [3], which are theorized to cause, or exacerbate, mental illness [4]. Psychological factors (e.g., weight stigma) may also explain the association [5]. Evidence from cross‐sectional and longitudinal studies indicates that body mass index (BMI) is strongly associated with depression [6], anxiety [7], eating disorders, and serious mental illness (i.e., bipolar disorder, schizophrenia, and psychosis) [8]. The distribution of adipose tissue in the body may also influence mental health. Abdominal adiposity produces a greater effect on systemic inflammation and homeostatic functions than other sites of excess adiposity and may have a stronger effect on health outcomes [9]. Indeed, co‐occurrence of mental illness with excess abdominal fat and metabolic disorders has been reported in epidemiological research [10].

However, previous evidence on the association between adiposity and mental illness may be confounded by reverse causation, bias, and residual confounding. Mental illness could lead to adiposity, partly through increased intake of (energy‐dense) food in response to negative emotions (e.g., emotional eating, low motivation for weight control), through metabolic changes (e.g. increased cortisol secretion), or as side effects of medications used to treat mental illness (i.e., weight gain) [11]. Moreover, obesity co‐occurs with many other risk factors, which can confound the association between obesity and mental illness. For example, socioeconomic deprivation, social adversity, reduced physical activity, and unhealthy diet are associated with both obesity and mental illness [12]. Therefore, assessing the causal association between adiposity and mental illness is challenging, but is nonetheless crucial to understanding disease etiology and to develop effective diagnostic, therapeutic, and preventative interventions. Such knowledge also has major public health implications because, despite the introduction of psychological therapy and medication, many patients respond poorly to these current treatments [13, 14], and many psychotherapeutic agents increase the risk of weight gain [15, 16].

Mendelian randomization (MR) analyses use genetic variants as instrumental variables, which— in principle— overcome the reverse causation and confounding that affects traditional or conventional observational study designs [17]. MR permits a robust assessment of the causal relationship between adiposity and mental illness. Recent MR studies have explored the causal role of adiposity in multiple mental illnesses, but results are yet to be summarized and assessed. We conducted a systematic review and meta‐analyses of MR studies to appraise and summarize evidence on causality between adiposity and mental illnesses.

## 2 Methods

This systematic review was conducted in accordance with the Preferred Reporting Items for Systematic Reviews and Meta‐Analysis (PRISMA) statement and the recent STROBE‐MR guideline [18, 19]. The protocol for the systematic review was prospectively registered with the International Prospective Register of Systematic Reviews (PROSPERO) on 31st May, 2022 (CRD42022336439).

### 2.1 Search Strategy

Searches were conducted on Embase, Medline, and Web of Science from database inception to September 22nd, 2025, using search terms that included synonyms for “adiposity” AND “mental illness” AND “Mendelian randomization.” This study was designed to include causal evidence that performed analyses referred to as “Mendelian randomization” (or closely related methods such as “genetic instrumental variable regression”), and the search terms used were detailed in Supplementary Material 1. We included studies that investigated the causal associations of adiposity (e.g., BMI, body fat percentage), abdominal adiposity (e.g., waist circumference, waist‐to‐hip ratio), peripheral adiposity (e.g., arm fat mass), and body composition (e.g., fat mass index) with depression, anxiety, eating disorders, bipolar disorder, obsessive compulsive disorder (OCD), post‐traumatic stress disorder (PTSD), schizophrenia, and other related disorders. The lead reviewer (MG) and one other reviewer (PJD, DAK, or YY) independently screened potentially eligible studies based on title and abstract, followed by full text where applicable.

### 2.2 Data Extraction

Data were extracted and entered in predefined data extraction tables independently by the lead reviewer (MG) and one of the other three reviewers (PJD, DAK, or YY) and finalized after discussion (Appendix S1 [20]). A third reviewer (PCD) was consulted in case of disagreement. For each included study, the following information was extracted: information on the exposure and outcome of interest, sample size, the genetic instrument, the MR design (one‐sample and/or two‐sample), population ancestry, and the MR analysis results. Following the MR dictionary [21], we defined one‐sample MR studies as those we interpreted as measuring the instrumental variable‐exposure and instrumental variable‐outcome associations in the same sample [22], and two‐sample MR studies as those which measured these associations in different samples [23]. Inverse variance weighted models were regarded as the main analysis [24, 25]. Individual‐level (usually associated with one‐sample MR) and summary‐level approaches (usually two‐sample MR) can be conducted in the same population. We extracted both sets of results where studies explicitly reported both one‐ and two‐sample results (e.g., [26]).

### 2.3 Quality Assessment

We developed a scoring system incorporating factors relating to the validity of MR studies that reflected the recently developed STROBE‐MR guidelines [19]. Important indices of quality are measurement of exposure and outcome (two items), selection of genetic variants (three items), and results of MR analysis (five items for one‐sample MR, seven items for two‐sample MR). Each item was given a score of “−,” “− +,”or “+” corresponding to high, moderate, or low risk of bias, respectively. For example, regarding the type of measure for the exposure, we rated this “−” if unclear or self‐reported, “− +” if a mix of self‐reported and measured, and “*+*” if objectively measured. Additional details are presented in Supplementary Material 2. Categories were given an overall score based on the most frequently occurring score, or the even score where two scores occurred at the same frequency. For example, for “selection of genetic variants,” which was measured by three items, we chose the modal score (i.e., the score that appeared most frequently) to indicate quality of selection of genetic variants; for scores that occurred at the same frequency, we chose the higher rounded even score; that is, “+” was chosen between “− +” and “+,”“− +” was chosen between “− +” and “−,” and “+ −” were chosen between “−” and “+.”For MR studies that did not describe *p*‐value threshold, we calculated this from the *F* statistic, % variance explained, or the variance from related MR studies that used similar/closely related sets of genetic variants as instruments. The lead reviewer (MG) and another reviewer (PJD, DAK, or YY) scored included studies independently, after which they compared their scores and any inconsistencies were resolved by discussion, or referral to a third reviewer (PCD).

### 2.4 Statistical Analysis

Most studies reported the odds ratio (OR) for a 1 standard deviation (*SD*) increase in adiposity indices, such as BMI. Where this was not the case, we rescaled the units of the outcome and corresponding standard errors to 1 *SD*. Where the unit of exposure was not reported, we assumed it to be 1 *SD*, as this is how MR studies with continuous exposure typically report outcomes when using adiposity‐related exposures. Exposures defined categorically were not included for meta‐analyses due to their inherent limitations in capturing the full spectrum of exposure data and potentially introducing bias. Where the exposure‐outcome was expressed as a regression coefficient for a continuous outcome, we used the Chinn equation to produce corresponding ORs to facilitate meta‐analysis [27]. Probit regression coefficients were converted into approximated logit coefficients by multiplying the probit coefficient by 1.6 [28]. We found that many included papers used the same cohorts to derive the genetic instruments, so when more than one MR estimate was published on the same outcome and study population, the MR estimate based on the largest number of cases, or if cases were identical, the largest number of genetic variants was retained for meta‐analysis. For outcomes reported as continuous or binary on the same scale (e.g., Patient Health Questionnaire‐9) among the same cohort, number of cases, and number of genetic instruments, we retained the binary clinical outcome for meta‐analysis (similar to Larsson & Burgess, 2021 [24]) (Supplementary Material 4).

Random‐effects models were used to produce summary effect sizes with 95% confidence intervals (CIs) from individual study data [29, 30]. The percentage of variability in effect estimates due to heterogeneity between studies was quantified using the *I* ^2^ statistic. For this, values < 25%, 25%–75%, and > 75% were considered to indicate low, moderate, and high heterogeneity, respectively [31]. Pre‐specified subgroup analyses by sex were conducted if sex‐specific estimates were provided. Where studies did not explicitly report ancestry, we examined the exposure and outcome genome‐wide association studies (GWASs) to infer likely ancestry. Sensitivity analyses were conducted to investigate the equivalence of a meta‐analysis of ORs and beta coefficients; thus, the main analysis was repeated including only studies reporting ORs with 95% CI and, separately, the main analysis with beta coefficients and 95% CI. All statistical analyses were conducted in Stata 14 SE (StataCorp, College Station, TX, United States) using the *metan* commands.

## 3 Results

The search strategy resulted in 4215 articles, of which 57 eligible MR studies with 215 estimates were included in this review (Figure 1; Supplementary Material 1). Among them, 30 studies reported one estimate [32, 33, 34, 35, 36, 37, 38, 39, 40, 41, 42, 43, 44, 45, 46, 47, 48, 49, 50, 51, 52, 53, 54, 55, 56, 57, 58, 59, 60, 61], six studies reported two estimates [62, 63, 64, 65, 66, 67], four studies reported three estimates [68, 69, 70, 71], four studies reported four estimates [72, 73, 74, 75], and 13 studies reported 5+ estimates [26, 76, 77, 78, 79, 80, 81, 82, 83, 84, 85, 86, 87]. Study characteristics and reported associations of the included studies are given in Table 1, and additional details are presented in Appendix S1 [20]. Forty‐one studies reported 117 estimates of the association between adiposity and depression [26, 33, 34, 35, 36, 38, 41, 43, 44, 45, 46, 48, 49, 51, 52, 54, 57, 58, 59, 60, 61, 62, 65, 67, 68, 69, 70, 71, 72, 73, 74, 75, 78, 79, 80, 82, 83, 84, 85, 86, 87], seven studies reported 14 estimates for anxiety [26, 42, 53, 56, 67, 83, 87], three studies reported eight estimates for anorexia nervosa [77, 83, 84], seven studies reported 15 estimates for bipolar disorder [32, 55, 65, 68, 70, 81, 83], four studies reported nine estimates for OCD [77, 83, 84, 87], three studies reported 23 estimates for PTSD [50, 76, 83], and 14 studies reported 29 estimates for schizophrenia [37, 39, 40, 47, 63, 64, 66, 68, 70, 77, 81, 83, 84, 87]. One‐sample MR design was conducted in six studies [34, 35, 44, 69, 72, 78], two‐sample MR design was conducted in 48 studies [32, 33, 36, 37, 38, 39, 40, 41, 42, 43, 45, 46, 47, 48, 49, 50, 51, 52, 53, 54, 55, 56, 57, 58, 59, 60, 61, 62, 63, 64, 65, 66, 67, 68, 70, 71, 73, 75, 76, 77, 79, 80, 81, 82, 83, 84, 85, 87], and both one‐sample MR and two‐sample MR were conducted in three studies [26, 74, 86]. Forty‐four unique MR estimates out of 215 total estimates were included in meta‐analyses (details as shown in “Yes” under “For meta‐analysis” column in Appendix S1 [20]).

**FIGURE 1:**
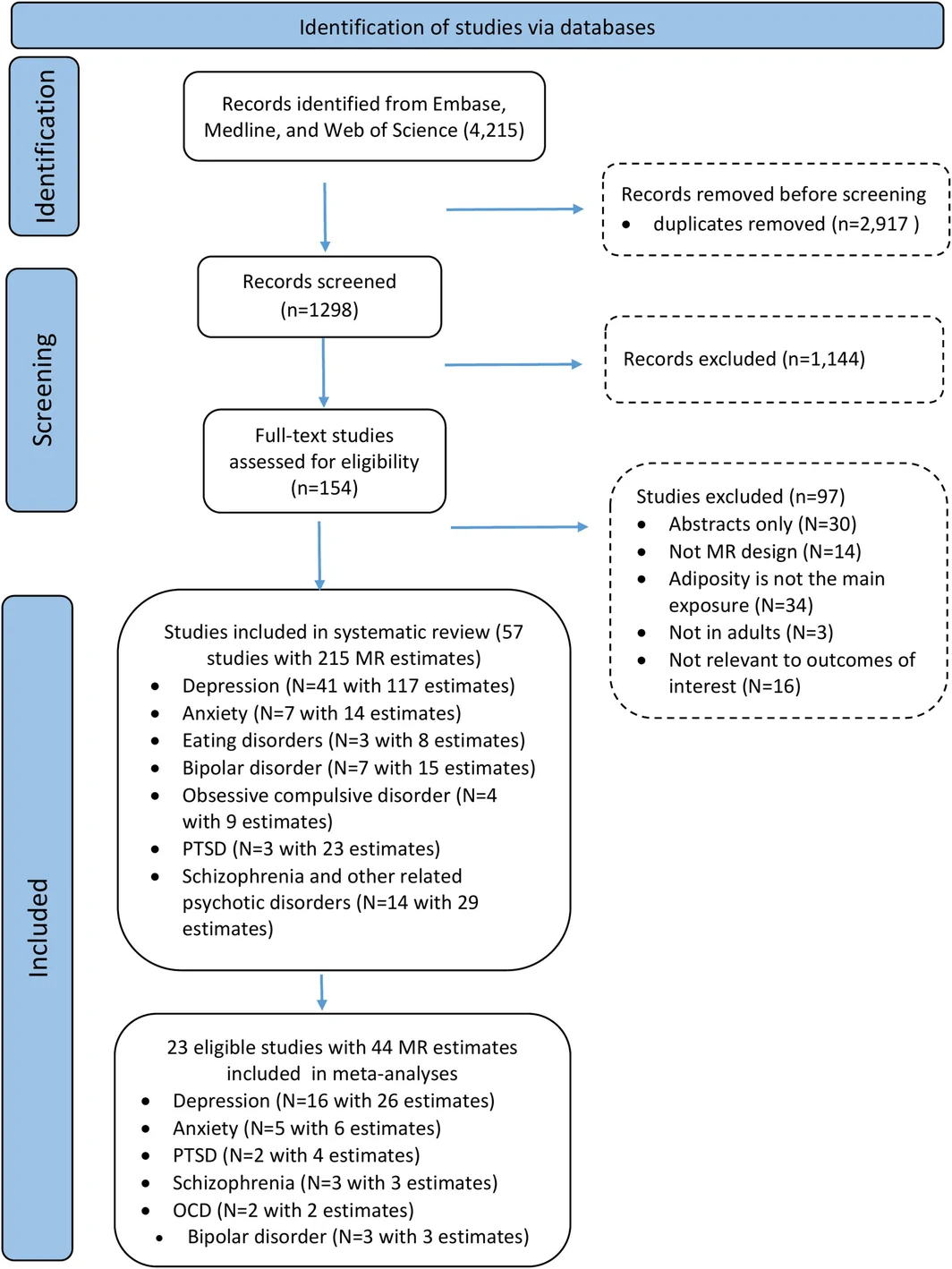
PRISMA flowchart of included studies and exclusion reasons.

**TABLE 1:** The characteristics of included Mendelian randomization studies of adiposity measures on mental illnesses.

Study	Exposure	Outcomes	Exposure source	Exposure data collection	Outcome source	Outcome data collection	MR design	Number of estimates	Ancestry
[72]	Adiposity (BMI)	Depression	GIANT	Self‐reported or measured	HRS, Add health	Self‐reported (by CES‐D)	One‐sample	4	European
[64]	Adiposity (BMI)	Schizophrenia	JENGER	Measured onsite	Ikeda 2019 and PGC Asia	Clinical diagnosis	Two‐sample	2	Asian/European
[51], [32]	Adiposity (BMI)	Bipolar disorder	UKB	Measured onsite	GWAS (Mullins et al., 2021)	Clinical diagnosis	Two‐sample	1	European
[52], [76]	Adiposity (BMI, body fat percentage, fat mass)	PTSD	UKB, GIANT	Self‐reported or measured	PGC‐PTSD (Nievergelt et al., 2019), PGC‐MVP (Stein et al., 2021)	Self‐report or clinical diagnosis	Two‐sample	20	European and African
	Abdominal adiposity (hip, waist, trunk fat percentage, trunk fat mass)	PTSD	UKB	Measured onsite	PGC‐PTSD (Nievergelt et al., 2019), PGC‐MVP (Stein et al., 2021)	Self‐report or clinical diagnosis	Two‐sample		European and African
	Peripheral adiposity (arm/leg fat mass and fat percentage)	PTSD	UKB	Measured onsite	PGC‐PTSD (Nievergelt et al., 2019), PGC‐MVP (Stein et al., 2021)	Self‐report or clinical diagnosis	Two‐sample		European and African
[26]	Adiposity (BMI)	Depression	UKB, GIANT	Self‐reported or measured	UKB	Self‐reported (CIDI‐SF and PHQ9)	One‐sample, two‐sample	19	European
	Adiposity (BMI)	Anxiety	UKB, GIANT	Self‐reported or measured	UKB	Self‐reported (GAD)	One‐sample, two‐sample		European
[83]	Adiposity (BMI)	Depression	GIANT	Self‐reported or measured	PGC (Howard, 2019)	Clinical diagnosis	Two‐sample	14	European
	Adiposity (BMI)	Anxiety	GIANT	Self‐reported or measured	PGC (Otowa, 2016)	Self‐report or clinical diagnosis	Two‐sample		European
	Adiposity (BMI)	PTSD	GIANT	Self‐reported or measured	PGC (Nievergelt, 2019)	Clinical diagnosis	Two‐sample		European
	Adiposity (BMI)	Anorexia nervosa	GIANT	Self‐reported or measured	PGC (Watson, 2019)	Clinical diagnosis	Two‐sample		European
	Adiposity (BMI)	Schizophrenia	GIANT	Self‐reported or measured	PGC (Trubetskoy, 2022)	Clinical diagnosis	Two‐sample		European
	Adiposity (BMI)	OCD	GIANT	Self‐reported or measured	PGC (Arnold, 2018)	Clinical diagnosis	Two‐sample		European
	Adiposity (BMI)	Bipolar disorder	GIANT	Self‐reported or measured	PGC (Mullins, 2021)	Clinical diagnosis	Two‐sample		European
	Abdominal adiposity (WHR)	Depression	GIANT	Self‐reported or measured	PGC (Howard, 2019)	Clinical diagnosis	Two‐sample		European
	Abdominal adiposity (WHR)	Anxiety	GIANT	Self‐reported or measured	PGC (Otowa, 2016)	Self‐report or clinical diagnosis	Two‐sample		European
	Abdominal adiposity (WHR)	Bipolar disorder	GIANT	Self‐reported or measured	PGC (Mullins, 2021)	Clinical diagnosis	Two‐sample		European
	Abdominal adiposity (WHR)	Anorexia nervosa	GIANT	Self‐reported or measured	PGC (Watson, 2019)	Clinical diagnosis	Two‐sample		European
	Abdominal adiposity (WHR)	OCD	GIANT	Self‐reported or measured	PGC (Arnold, 2018)	Clinical diagnosis	Two‐sample		European
	Abdominal adiposity (WHR)	PTSD	GIANT	Self‐reported or measured	PGC (Stein, 2021)	Clinical diagnosis	Two‐sample		European
	Abdominal adiposity (WHR)	Schizophrenia	GIANT	Self‐reported or measured	PGC (Trubetskoy, 2022)	Clinical diagnosis	Two‐sample		European
[75], [33]	Adiposity (BMI)	Depression	GIANT	Self‐reported or measured	23andMe	Self‐report or clinical diagnosis	Two‐sample	1	European
[84]	Adiposity (Class I obesity, Class II obesity, Class III obesity)	Depression	Berndt et al., 2013	Self‐reported or measured	PGC (Howard)	Clinical diagnosis	Two‐sample	10	European
	Adiposity (Class I obesity, Class II obesity, Class III obesity)	Anorexia nervosa	Berndt et al., 2013	Self‐reported or measured	PGC (Watson)	Clinical diagnosis	Two‐sample		European
	Adiposity (Class I obesity, Class II obesity, Class III obesity)	Schizophrenia	Berndt et al., 2013	Self‐reported or measured	PGC (Pardinas)	Clinical diagnosis	Two‐sample		European
	Adiposity (Class I obesity, Class II obesity, Class III obesity)	OCD	Berndt et al., 2013	Self‐reported or measured	PGC (Arnold)	Clinical diagnosis	Two‐sample		European
[53, 54], [68]	Adiposity (BMI)	Depression	GIANT	Self‐reported or measured	PGC (Ripke,2013)	Clinical diagnosis	Two‐sample	3	European
	Adiposity (BMI)	Bipolar disorder	GIANT	Self‐reported or measured	PGC (Sklar,2011)	Clinical diagnosis	Two‐sample		European
	Adiposity (BMI)	Schizophrenia	GIANT	Self‐reported or measured	PGC (Ripke,2014)	Clinical diagnosis	Two‐sample		European
[65]	Adiposity (BMI)	Depression	UKB	Measured onsite	PGC (Howard,2019)	Self‐report or clinical diagnosis	Two‐sample	2	European
	Adiposity (BMI)	Bipolar disorder	UKB	Measured onsite	PGC (Stahl,2019)	Clinical diagnosis	Two‐sample		European
2023 [48]	Adiposity (BMI)	Depression	GIANT	Self‐reported or measured	PGC (Howard) without 23andMe	Self‐report or clinical diagnosis	Two‐sample	1	European
[70], [77]	Adiposity (BMI, body fat percentage, fat mass)	Anorexia nervosa	UKB	Measured onsite	GWAS (Watson,2019)	Self‐report or clinical diagnosis	Two‐sample	9	European
	Adiposity (BMI, body fat percentage, fat mass)	Obsessive compulsive disorder	UKB	Measured onsite	GWAS (Mattheisen,2015)	Clinical diagnosis	Two‐sample		European
	Adiposity (BMI, body fat percentage, fat mass)	Schizophrenia	UKB	Measured onsite	PGC (Ripke,2014)	Clinical diagnosis	Two‐sample		European
[34]	Adiposity (BMI)	Depression	Speliotes et al	Self‐reported or measured	RADIANT study	Clinical diagnosis	One‐sample	1	European
[35]	Adiposity (BMI)	Depression	Young Finns	Measured onsite	Young Finns	Self‐reported (by BDI)	One‐sample	1	European
[36]	Adiposity (BMI)	Depression	GIANT	Self‐reported or measured	PGC (Howard et al., 2019)	Self‐report or clinical diagnosis	Two‐sample	1	European
[69]	Adiposity (BMI)	Depression	UKB	Measured onsite	UKB	Clinical diagnosis	One‐sample	3	European
[78]	Adiposity (BMI)	Depression	UKB	Measured onsite	UKB	Self‐reported (few questions)	One‐sample	6	European
	Abdominal adiposity (WHR)	Depression	GIANT	Self‐reported or measured	UKB	Self‐reported (few questions)	One‐sample		European
[88]	Adiposity (BMI)	Schizophrenia	GIANT	Self‐reported or measured	PGC (Trubetskoy,2022)	Clinical diagnosis	Two‐sample	2	European
	Abdominal adiposity (WHR)	Schizophrenia	GIANT	Self‐reported or measured	PGC (Trubetskoy,2022)	Clinical diagnosis	Two‐sample		European
[62]	Adiposity (BMI)	Depression	Whitehall II	Measured onsite	Whitehall II	Self‐reported (by GHQ)	Two‐sample	2	Primarily European
[79]	Adiposity (BMI)	Depression	CGPS	Measured onsite	CGPS	Self‐reported	Two‐sample	7	European (Danish)
	Abdominal adiposity (WHR)	Depression	CGPS	Measured onsite	CGPS	Self‐reported	Two‐sample		European (Danish)
[55, 71], [73]	Adiposity (BMI, body fat percentage)	Depression	GIANT	Self‐reported or measured	FinnGen, PGC (Wray et al., 2018)	Hospital discharge register and/or causes of death register	Two‐sample	4	European
[85]	Adiposity (BMI)	Depression	GIANT, UKB	Self‐reported or measured	African Summary data, East Asian Summary data, GWAS (Meng), Hispanic/Latin American Summary data, South Asian Summary data	Clinical diagnosis	Two‐sample	5	European
[86]	Adiposity (BMI)	Depression	GIANT, UKB, CKB	Self‐reported or measured	CKB	Self‐report or clinical diagnosis	One‐sample, two‐sample	6	East Asian
	Abdominal adiposity (WHR)	Depression	GIANT, UKB, CKB	Self‐reported or measured	CKB	Self‐report or clinical diagnosis	One‐sample, two‐sample		East Asian
[49]	Adiposity (BMI)	Depression	GIANT, UKB, CKB	Self‐reported or measured	PGC&23andMe	Self‐report or clinical diagnosis	Two‐sample	1	European
[67], [63]	Adiposity (BMI)	Schizophrenia	GIANT	Self‐reported or measured	PGC (Pardinas, 2018)	Clinical diagnosis	Two‐sample	2	Primarily European
[37]	Abdominal adiposity (WHR)	Schizophrenia	GWAS (Pulit et al., 2019)	Self‐reported or measured	PGC (Pardinas, 2018)	Clinical diagnosis	Two‐sample	1	European
[80]	Adiposity (BMI)	Depression	GWAS (Yengo. et al., 2018)	Self‐reported or measured	CoLaus|PsyCoLaus, NESDA|NTR, C|P|N| N	Diagnostic Interview	Two‐sample	12	European
	Abdominal adiposity (WHR)	Depression	GWAS (Pulit et al., 2019)	Self‐reported or measured	CoLaus|PsyCoLaus, NESDA|NTR, C|P|N| N	Diagnostic Interview	Two‐sample		European
[50]	Abdominal adiposity (WHR)	PTSD	GIANT	Self‐reported or measured	PGC‐PTSD (Duncan, 2018)	Clinical diagnosis	Two‐sample	1	European
[38]	Adiposity (BMI)	Depression	GWAS (Yengo. et al., 2018)	Self‐reported or measured	PGC (Wray, 2018)	Self‐report or clinical diagnosis	Two‐sample	1	European
[56], [39]	Adiposity (BMI)	Schizophrenia	GIANT	Self‐reported or measured	PGC (Ripke,2014)	Clinical diagnosis	Two‐sample	1	Primarily European
[40]	Adiposity (BMI)	Schizophrenia	UKB	Measured onsite	PGC (Ripke, 2014)	Clinical diagnosis	Two‐sample	1	European
[81]	Adiposity (BMI, body fat percentage)	Bipolar disorder	GIANT, GWAS (Lu et al., 2016)	Self‐reported or measured	GWAS (Hou, 2016)	Interview and clinical diagnosis	Two‐sample	16	Primarily European
	Adiposity (BMI, body fat percentage)	Schizophrenia	GIANT, GWAS	Self‐reported or measured	PGC (Ripke, 2013)	Clinical diagnosis	Two‐sample		Primarily European
	Abdominal adiposity (WHR)	Bipolar disorder	GIANT	Measured onsite	GWAS (Hou, 2016)	Interview and clinical diagnosis	Two‐sample		Primarily European
	Abdominal adiposity (WHR)	Schizophrenia	GIANT	Measured onsite	PGC (Ripke, 2013)	Clinical diagnosis	Two‐sample		Primarily European
	Adipose tissue	Bipolar disorder	GWAS (Chu et al., 2017)	Measured onsite	GWAS (Hou, 2016)	Interview and clinical diagnosis	Two‐sample		Primarily European
	Adipose tissue	Schizophrenia	GWAS (Chu et al., 2017)	Measured onsite	GWAS (Hou, 2016)	Clinical diagnosis	Two‐sample		Primarily European
[82]	Adiposity (BMI, fat mass, body fat percentage)	Depression	UKB	Measured onsite	PGC (Wray, 2018)	Self‐report or clinical diagnosis	Two‐sample	13	European
	Abdominal adiposity (trunk fat percentage, trunk fat mass)	Depression	UKB	Measured onsite	PGC (Wray, 2018)	Self‐report or clinical diagnosis	Two‐sample		European
	Peripheral adiposity (arm/leg fat mass and fat percentage)	Depression	UKB	Measured onsite	PGC (Wray, 2018)	Self‐report or clinical diagnosis	Two‐sample		European
[74]	Adiposity (BMI)	Depression	UKB	Measured onsite	UKB, PGC (Wray, 2018)	Self‐report or clinical diagnosis	One‐sample, two‐sample	4	European
[41]	Adiposity (BMI)	Depression	GIANT	Self‐reported or measured	23andMe	Clinical diagnosis	Two‐sample	1	European
[44]	Adiposity (BMI)	Depression	Speliotes et al	Self‐reported or measured	Add Health	Self‐reported (by CES)	One‐sample	1	European and African
[42]	Adiposity (BMI)	Anxiety	Speliotes et al	Self‐reported or measured	NHS and HPFS	Self‐reported (CCI)	Two‐sample	1	Primarily European
[43]	Adiposity (BMI)	Depression	Speliotes et al	Self‐reported or measured	NHS	Self‐reported (GDS)	Two‐sample	1	Primarily European
[46]	Adiposity (BMI)	Depression	GIANT	Self‐reported or measured	Multiple	Self‐reported and clinical diagnosis	Two‐sample	1	European
[47, 57, 87]	Adiposity (BMI)	Schizophrenia	UKB	Measured onsite	PGC (Trubetskoy et., al 2022)	Clinical diagnosis	Two‐sample	1	European
[45, 58, 59]	Abdominal adiposity (waist)	Depression	GIANT	Self‐reported or measured	UKB and PGC	Self‐report or clinical diagnosis	Two‐sample	1	Primarily European

*Note:* Some studies/estimates were used in meta‐analysis because of the rules for avoiding overlapping samples/picking the studies with the largest N of genetic variants as set out in the Methods section [60, 61].

Abbreviations: Add Health, National Longitudinal Study of Adolescent to Adult Health; BMI, body mass index; C|P|N|N, CoLaus|PsyCoLaus combined NESDA|NTRBMI: Body mass index; CCI, the validated phobic anxiety subscale of the Crown Crisp Index (CCI); CKB, China Kadoorie Biobank; CES‐D, Center for Epidemiologic Studies Depression Scale (CES‐D); CGPS, Copenhagen General Population Study; CIDI‐SF, Composite International Diagnostic Interview Short Form; JENGER, Biobank Japan; GAD, Generalized Anxiety Disorder Assessment (GAD‐7); GDS, Geriatric Depression Scale 15 (GDS‐15); GIANT, The Genetic Investigation of ANthropometric Traits consortium; GWAS, Genome‐wide association study; HPFS, Nurses' Health Study (NHS) and Health Professionals Follow‐up Study; HRS, Health and Retirement Study; NHS, Nurses' Health Study; PGC, Psychiatric Genomics Consortium; PHQ9, Patient Health Questionnaire‐9; UKB, UK Biobank; WHR, waist‐to‐hip ratio; Young Finns, Cardiovascular Risk in Young Finns; 23andMe, also named Social Science Genetic Association Consortium; Multiple: PGC29 (29 samples of European ancestry), deCODE, GenScotland (Generation Scotland: Scottish Family Health Study), GERA (Genetic Epidemiology Research on Adult Health and Aging cohort), iPSYCH (Integrative Psychiatric Research consortium), UK Biobank and 23andMe.

### 3.1 Quality Assessment of Included Studies

Quality assessment of the included studies is presented in Table 2 and Supplementary Material 3. Among the 51 studies using two‐sample MR design, 45 studies scored “+” in the analysis category [26, 32, 33, 36, 37, 38, 39, 40, 41, 45, 46, 47, 48, 49, 50, 51, 52, 53, 54, 55, 57, 58, 59, 60, 61, 63, 64, 65, 66, 67, 68, 70, 71, 73, 74, 76, 77, 80, 81, 82, 83, 84, 85, 86, 87] and only six studies scored “−” in the analysis category [42, 43, 56, 62, 75, 79], meaning that most studies tested the majority of bidirectional effects, assessment of nonlinearity for continuous exposures, heterogeneity, reported data on pleiotropy‐robust sensitivity analysis, similar results among all MR analyses, and for two‐sample MR SNP harmonization across samples, and estimates of SNP‐exposure and SNP‐outcome from comparable populations. Among the nine included studies using a one‐sample MR design, two studies scored “+” in the analysis category [69, 86], with the remaining seven studies scoring “−” [26, 34, 35, 44, 72, 74, 78].

**TABLE 2:** Methodological quality assessment of the included Mendelian randomization studies.

Author	Measurement of exposure and outcome	Selection of genetic variants	Analysis (one‐sample MR)	Analysis (two‐sample MR)
Amin 2020 [71]	**− +**	**+**	**−**	
Aoki 2022 [63]	**+**	**−**		**+**
Ardissino 2024 [50]	**+**	**+**		+
Byg 2022 [31]	**+**	**− +**		**+**
Cai 2024 [51]	− +	+		+
Carvalho 2021 [75]	**− +**	**+**		**+**
Casanova 2021 [25]	**− +**	**+**	**−**	**+**
Chen 2023 [82]	**− +**	**+**		**+**
Chen 2025 [74]	− +	−		**−**
Day 2018 [32]	**− +**	**+**		**+**
Ding 2022 [83]	**+**	**+**		**+**
Fan 2025 [52]	**− +**	+		+
Guo 2024 [53]	+	−		+
Hartwig 2016 [67]	**+**	**+**		**+**
He ML 2023 [64]	**+**	**+**		**+**
He RX 2023 [47]	**− +**	**+**		**+**
Huang 2024 [69]	+	+		+
Hubel 2019 [76]	**+**	**−**		**+**
Hung 2014 [33]	**+**	**− +**	**−**	
Jokela 2012 [34]	**+**	**− +**	**−**	
Kappelmann 2021 [35]	**− +**	**+**		**+**
Karageorgiou 2023 [68]	**+**	**+**	**+**	
Khandaker 2020 [77]	**− +**	**+**	**−**	
Khani 2023 [65]	**+**	**−**		**+**
Kivimaki 2011 [61]	**+**	**−**		**−**
Lawlor 2011 [78]	**− +**	**−**		**−**
Luo 2024 [70]	− +	+		+
Ma 2025 [54]	+	+		+
Martin 2022 [72]	**+**	**+**		**+**
Meng 2024 [84]	**− +**	**−**		**+**
O'Loughlin 2023 [85]	**− +**	**+**	**+**	**+**
Palmos 2023 [48]	**− +**	**+**		**+**
Pathak 2025 [66]	+	+		+
Perry 2021 [62]	**+**	**+**		**+**
Peters 2020 [36]	**+**	**− +**		**+**
Pistis 2021 [79]	**+**	**+**		**+**
Polimanti 2017 [49]	**+**	**+**		**+**
Qi 2019 [37]	**− +**	**−**		**+**
Qin 2025 [55]	+	+		−
Rees 2019 [38]	**+**	**+**		**+**
Richardson 2019 [39]	**+**	**−**		**+**
So 2019 [80]	**+**	**+**		**+**
Speed 2019 [81]	**+**	**+**		**+**
Tyrrell 2019 [73]	**+**	**+**	**−**	**+**
vandenBroek 2018 [40]	**+**	**− +**		**+**
Walter 2015a [41]	**− +**	**−**		**−**
Walter 2015b [42]	**− +**	**−**		**−**
Willage 2018 [43]	**− +**	**−**	**−**	
Wray 2018 [45]	**− +**	**−**		**+**
Xiao 2024 [86]	+	+		+
Yang 2025 [56]	+	+		+
Yu 2023 [46]	**+**	**+**		**+**
Yuan 2025 [57]	+	+		+
Zhan 2025 [58]	+	+		+
Zhang 2021 [44]	**− +**	**−**		**+**
Zhang 2024 [59]	+	+		+
Zhou 2025 [60]	− +	+		+

*Note:* Green color indicates low risk of bias, yellow color indicates moderate risk of bias, pink color indicates high risk of bias, and gray color indicates not applicable.

### 3.2 Meta‐Analyses Results for General Adiposity and Mental Illnesses

Fifty‐two studies with 152 estimates provided data for the association between general adiposity and mental illnesses [26, 32, 33, 34, 35, 36, 38, 39, 40, 41, 42, 43, 44, 46, 47, 48, 49, 51, 52, 53, 54, 55, 56, 58, 60, 61, 62, 63, 64, 65, 66, 67, 68, 69, 70, 71, 72, 73, 74, 75, 76, 77, 78, 79, 80, 81, 82, 83, 84, 85, 86, 87], and 23 studies with 35 estimates were included in meta‐analysis [34, 35, 42, 43, 49, 53, 55, 58, 60, 62, 64, 67, 70, 72, 75, 76, 79, 80, 83, 85, 86, 87], as shown in Figure 2A,B. Most studies presented data on the association with depression. There was generally consistent, but imprecise, evidence that genetically predicted higher adiposity was associated with an increased risk of depression, with a combined OR of 1.09 (95% CI 1.02–1.15, *n* = 19 estimates) per SD of general adiposity, but with high heterogeneity (*I* ^2^ = 94%) between studies. However, there was no evidence of a causal association between genetically predicted adiposity and anxiety (OR 1.06, 95% CI 0.99–1.13, *n* = 6 estimates) [26, 42, 53, 67, 87], PTSD (OR 1.15, 95% CI 0.87–1.54, *n* = 2 estimates) [76, 83], or bipolar disorder (OR 0.99, 95% CI 0.91–1.07, *n* = 3 estimates) [55, 70, 83]. Limited evidence indicated that genetically predicted higher adiposity was associated with a reduced risk of schizophrenia (OR 0.83, 95% CI 0.77–0.90, *n* = 3 estimates) [64, 83, 87] and OCD (OR 0.69, 95% CI 0.59–0.80, *n* = 2 estimates) [83, 87]. Most of these analyses showed high heterogeneity.

**FIGURE 2:**
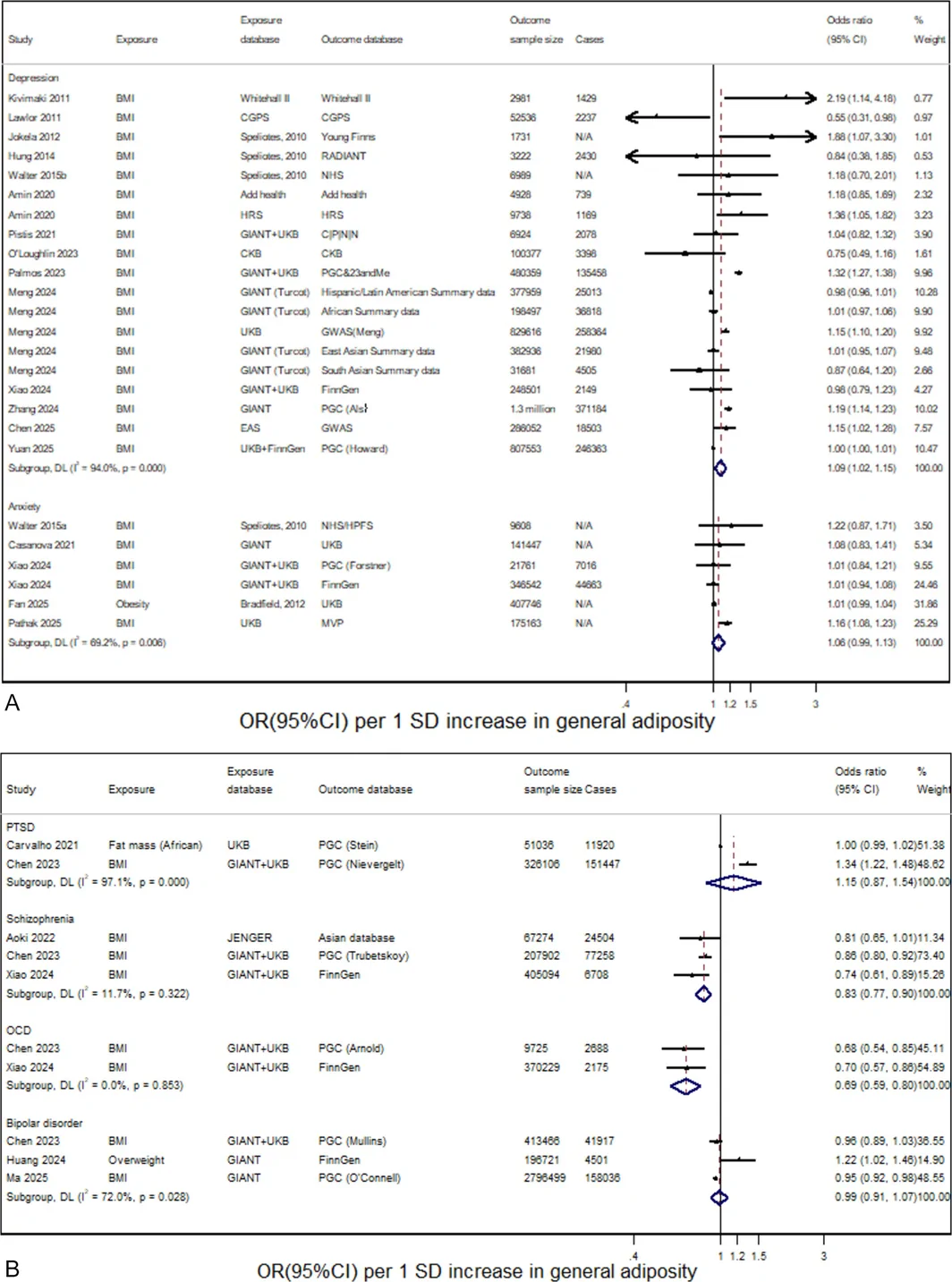
(A) Meta‐analysis results for genetically predicted general adiposity in relation to depression and anxiety. (B) Meta‐analysis results for genetically predicted general adiposity in relation to PTSD, schizophrenia, OCD, and bipolar disorder.

### 3.3 Meta‐Analyses Results of Abdominal Adiposity With Mental Illnesses

Fourteen studies with 38 estimates reported the causal relationship between abdominal adiposity and mental illness [37, 45, 50, 57, 59, 66, 76, 78, 79, 80, 81, 82, 83, 86], and seven studies with nine estimates were included in meta‐analyses after excluding overlapping study populations [57, 59, 76, 79, 80, 83, 86], as shown in Figure 3. There was no evidence of a causal association between abdominal adiposity and depression (OR 1.01, 95% CI 0.93–1.09, *n* = 7 estimates) [57, 59, 79, 80, 83, 86] or abdominal adiposity and PTSD (OR 1.12, 95% CI 0.93–1.34, *n* = 2 estimates) [76, 83].

**FIGURE 3:**
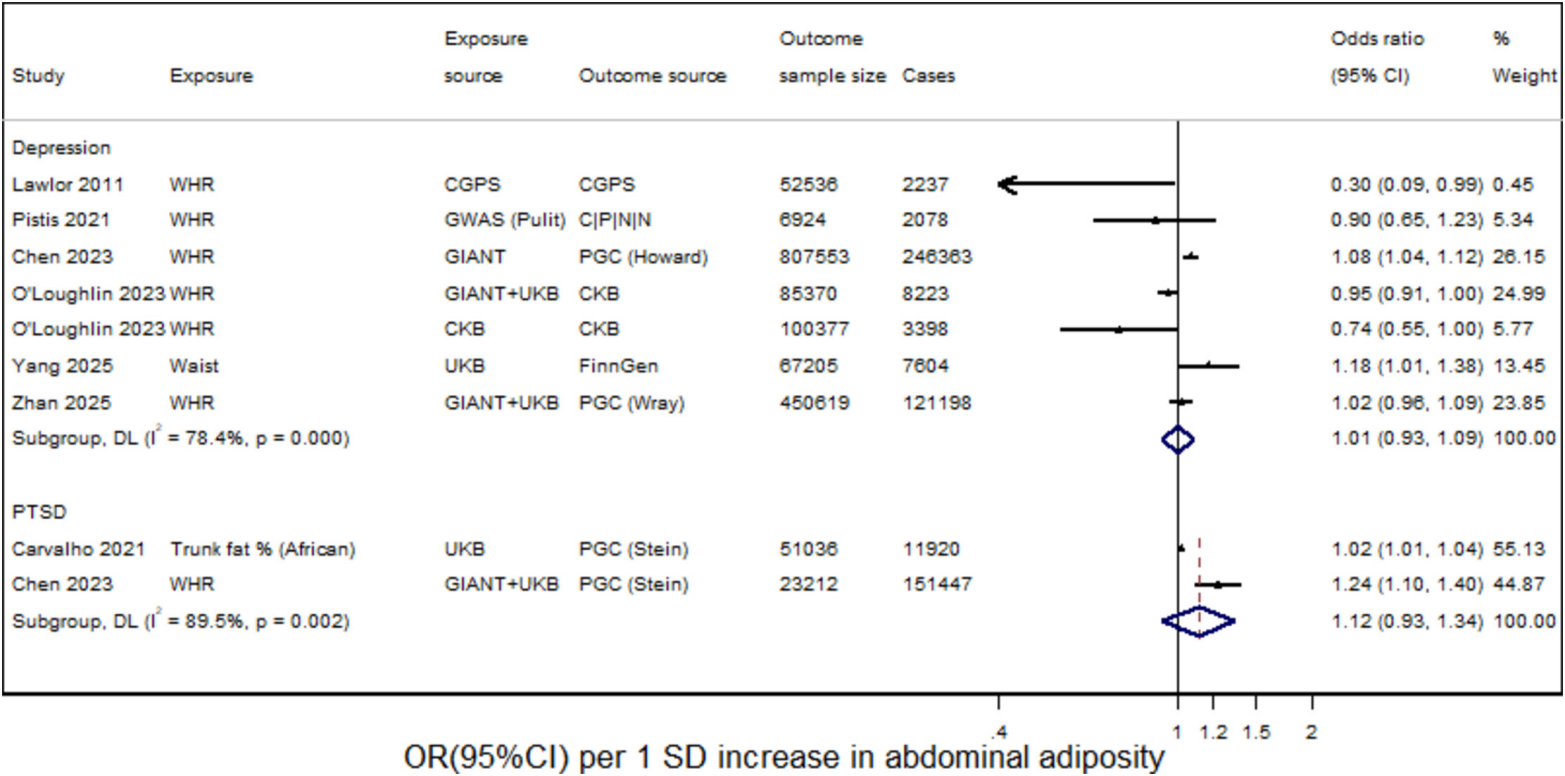
Meta‐analysis results for genetically predicted abdominal adiposity in relation to depression and PTSD.

### 3.4 Description of the Associations Between Adiposity Indices and Other Mental Illnesses

One study with six estimates on the associations of adipose tissue and bipolar disorder and schizophrenia was not included for meta‐analyses because estimates were derived from a single cohort (Appendix S1 [20]) [81]. For general adiposity, three studies examined the relationship with anorexia nervosa [77, 83, 84], with the largest study reporting a risk of 1.43 (95% CI 1.30–1.58) [83]. For general adiposity and OCD, the largest study reported a risk estimate of 0.68 (95% CI 0.54–0.85) [83]. For peripheral adiposity, two studies with 17 estimates reported the causal relationship between peripheral adiposity and mental illnesses [76, 82]. Associations for major depression were not included in the meta‐analysis due to overlapped cohorts. However, most estimates demonstrated significant causal associations [82]. Associations for PTSD were not included in the meta‐analysis because this study only reported significant results from European ancestry; results from African ancestry were eligible but not included in the meta‐analysis due to overlapped cohorts. For abdominal adiposity, one study reported the associations of abdominal adiposity with anxiety (1.23 [95% CI 0.98–1.56]), anorexia nervosa (1.29 [95% CI 1.15–1.45]), and OCD (0.59 [95% CI 0.44–0.78]) [83].

### 3.5 Subgroup Analyses

Pre‐specified subgroup analyses were performed to estimate the associations of genetically predicted adiposity measures with mental illnesses stratified by sex. In Appendix S1 [20], eight studies with 77 estimates provided the associations of genetically predicted BMI with depression and anxiety by sex [26, 44, 51, 72, 74, 86]. There was no evidence from meta‐analysis including 19 MR estimates that the causal relationship between BMI and depression differed by sex (*p* = 0.318; Figure 4A). Similar results were also found for anxiety (*p* = 0.287) as shown in Figure 4B [26, 42].

**FIGURE 4:**
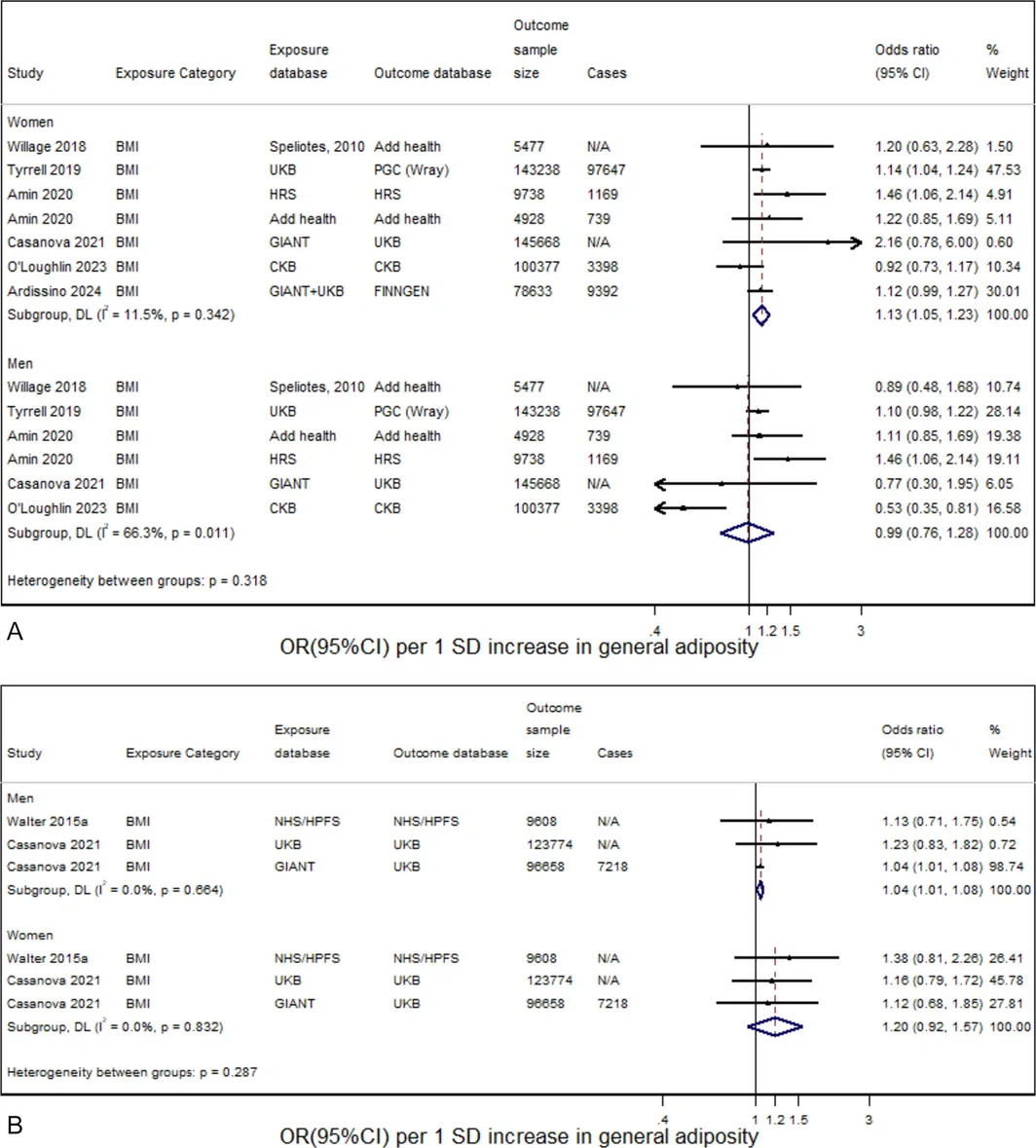
(A) Meta‐analysis results for genetically predicted adiposity in relation to depression stratified by sex. (B) Meta‐analysis results for genetically predicted adiposity in relation to anxiety stratified by sex.

### 3.6 Sensitivity Analyses for Clinical Diagnosis of Depression

We repeated the main analyses estimating the association of genetically predicted adiposity with depression, including only studies that reported results with binary outcomes. In Appendix S1 [20], 12 studies with 17 estimates provided results with binary outcomes (OR 1.08, 95% CI 1.02–1.15). The findings were similar to the main analysis, which included binary and continuous outcomes (Figure 5).

**FIGURE 5:**
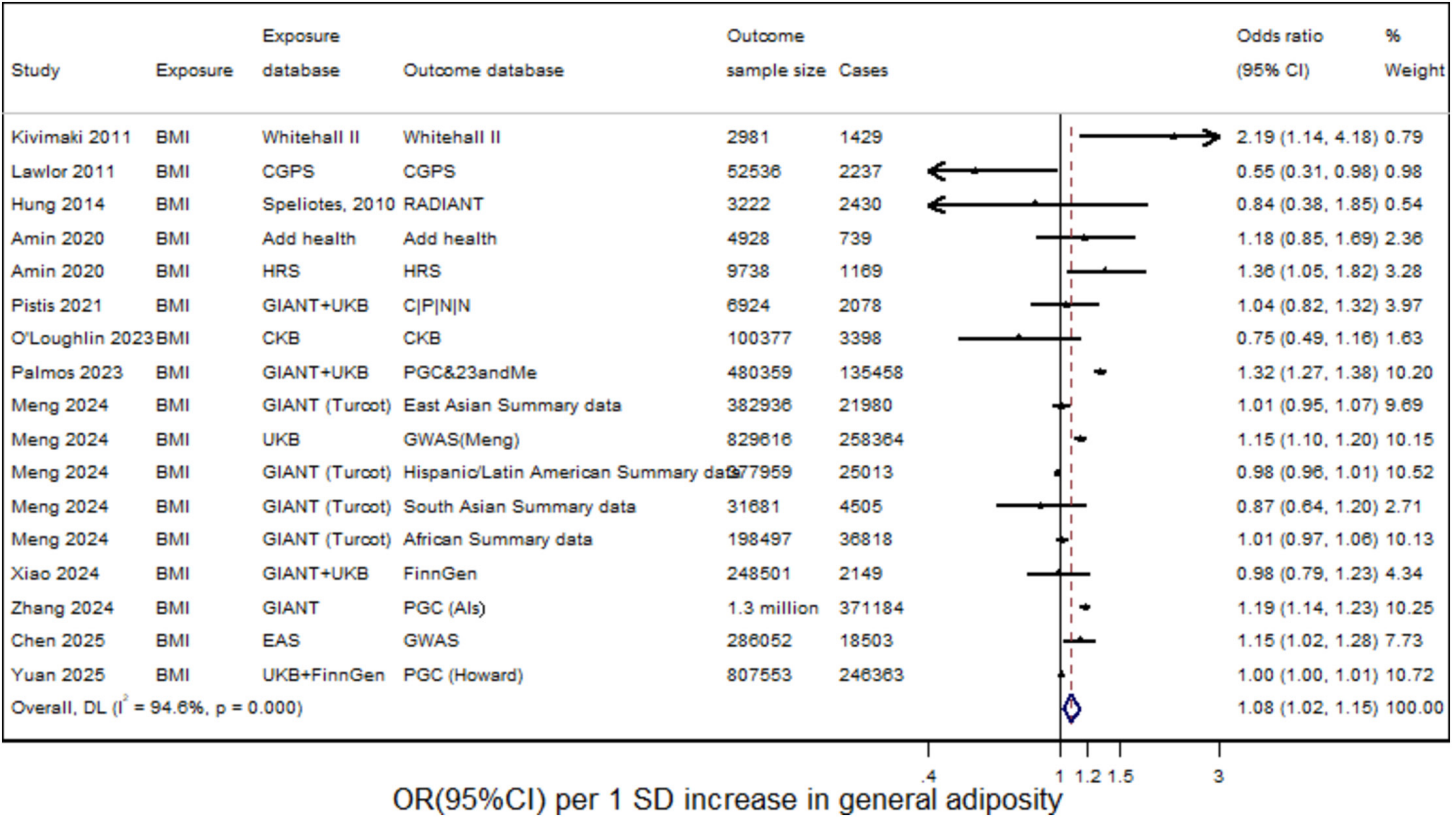
Meta‐analysis results for adiposity with depression in studies that reported odds ratios.

## 4 Discussion

This systematic review and meta‐analysis of MR studies regarding adiposity and mental illnesses found a causal relationship between general adiposity and depression, although effect sizes were modest. No sex difference was observed in this causal relationship. There was suggestive evidence for associations between different measures of adiposity and schizophrenia and OCD, while the evidence for PTSD, bipolar disorder, and anorexia nervosa was uncertain. The findings from this review and meta‐analysis are timely and address an important evidence gap. Presently, 39% of adults worldwide live with obesity and 12.5% mental illness, respectively [89, 90]; our findings suggest that higher general adiposity can cause depression.

A previous systematic review of eight MR studies on obesity and depression with last literature searches in October 2021 found consistent evidence, with a higher depression risk associated with per SD in BMI (OR: 1.33, 95% CI 1.19–1.48) [91]. Compared with that review, we selected studies with the largest GWAS data available for more types of mental illnesses and avoided selecting the same or overlapping cohorts where larger studies were available. This accounts for a large proportion of MR associations that were not included for meta‐analysis. For example, we selected studies that utilized the PGC (Howard) dataset for depression over those using the PGC (Wray) dataset (Supplementary material 4). Our results are in line with a meta‐analysis of randomized trials of behavioral weight loss programs that found modest improvements in depression at 12 months from baseline, but with insufficient data for symptoms of other mental illnesses [92].

Apart from depression, the causal effect of adiposity on other mental illnesses has been less studied using MR study designs. These findings are less certain but do not exclude meaningful causal associations. Meta‐analyses from conventional epidemiological research designs reported that obesity is associated with an increased likelihood of anxiety disorder (OR 1.30, 95% CI 1.20–1.41) [93] and an increased prevalence of bipolar disorder (OR 1.77, 95% CI 1.40–2.23) [94]. In our meta‐analyses, we did not find evidence of a causal role of adiposity in anxiety, PTSD, bipolar disorder, or anorexia nervosa, but data were imprecise, so more data would aid in further elucidating these relationships. Similarly, the small number of MR studies that assessed evidence of a causal relationship specifically between abdominal adiposity and mental illnesses, including depression, produced imprecise evidence compatible with either no risk or moderate‐sized increased risks. Conventional study designs have indicated that fat distribution (abdominal adiposity vs. general adiposity) is associated with depressive symptoms independent of general adiposity [95]. Given the small number of MR studies that reported abdominal adiposity and mental illness, further MR research with different and larger samples is needed to explore the causality of abdominal adiposity for mental illnesses.

Assessing the quality of analyses in MR is challenging due to the complexity and interrelationships between different elements of study design and the type of analysis used. A limitation of our review is that we attempted to characterize some of the main sources of possible bias, but arguably, we did not characterize all potential sources. For example, it was difficult, given reporting standards in some of the included literature, to fully characterize any sample overlap and the extent to which this may have biased two‐sample analysis. A similar issue arose with characterizing the genetic ancestries included in each study. To guide our quality assessment, we developed a checklist that reflected STROBE MR guidance. Using this quality assessment tool, we observed differences in methodological quality between the included studies. We focused on potential violations of the instrumental variable assumptions that underlie MR analyses, particularly the quality of analysis reported in each study. For example, a critical issue that may introduce bias is violations of the exclusion restriction due to pleiotropy. Newer studies tended to perform better when assessed against criteria relating to the quality of analysis, whereas older studies were undertaken before methods for pleiotropy sensitivity analysis were fully developed. Nevertheless, the overall conclusions of the meta‐analysis should be considered alongside the quality assessment, especially regarding the adequacy of pleiotropy evaluation in individual studies.

This review has several other limitations. First, statistical heterogeneity in most meta‐analyses was moderate to high. This suggests that more evidence may be needed to investigate or strengthen the causal association of more indices of adiposity with mental illness. Second, selection bias should be considered. For example, participants from cohorts such as the UK Biobank are relatively healthier than the general population, and studies relying on evidence from volunteer cohorts of healthy and relatively affluent populations may be affected by collider bias associated with self‐selection [96]. Third, although these analyses provide evidence for the causal relationship between general adiposity and depression, we were unable to assess the mechanisms that may underlie this causal relationship. Fourth, most studies pertained to populations of predominantly European ancestry. Fifth, as noted, our quality assessment did not capture all aspects of potential bias in MR studies. For example, we did not identify studies that relied wholly on within‐family designs, which may provide more robust estimates of causal associations in the presence of dynastic biases and cryptic population stratification [97]. We did not formally assess studies for whether they relied on samples of unrelated individuals, and this appears to be an area where reporting could be improved in future MR research.

## 5 Conclusions

Adiposity appears to be causally associated with depression. Evidence for associations with other mental health phenotypes remains suggestive or unclear. Future studies are needed to elucidate the mechanistic causal links between adiposity and depression.

## Author Contributions

**M.G.:** conceptualization, methodology, software, formal analysis, investigation, writing – original draft, writing – review and editing. **P.J.D.:** methodology, investigation, writing – review and editing. **D.A.K.:** methodology, investigation, writing – review and editing. **Y.Y.:** investigation, writing – review and editing. **P.A.:** conceptualization, methodology, writing – review and editing. **R.S.:** statistical methodology, writing – review and editing. **P.C.D.:** methodology, investigation, writing – review and editing, supervision. **M.G.** and **P.C.D.** are guarantors of the study.

## Conflicts of Interest

The authors declare no conflicts of interest.

## Supporting information


**Data S1:** Supporting Information.

**Appendix S1:** Supporting Information.

## Data Availability

All the data supporting the conclusions of this article are included within the article and its supporting information.
