# Supplementary material for: The Causal Role of Adiposity in Mental Illness: A Systematic Review and Meta‐Analysis of Mendelian Randomization Studies
Source: Obes Rev. 2026 May 19;27(12):e70156. doi: 10.1111/obr.70156 (PMC13749290; doi:10.1111/obr.70156)
Supplement: Supplementary file 1 [file OBR-27-e70156-s002.pdf]

The causal role of adiposity in mental illness: A systematic review and meta-analysis of Mendelian randomization studies

Authors

Min Gao [1,2,5*], Paul J Doody [1,2,6], Dimitrios A Koutoukidis [1,3], Yebo Yu [4], Paul Aveyard [1,2,3,5], Richard Stevens [1.2], Padraig C Dixon [1*]

Affiliations

[1] University of Oxford, Nuffield Department of Primary Care Health Sciences, Radcliffe Observatory Quarter, Oxford, UK

[2] NIHR Applied Research Collaboration Oxford and Thames Valley, Oxford University Hospitals, NHS Foundation Trust, Oxford, UK

[3] NIHR Oxford Biomedical Research Centre, Oxford University Hospitals, NHS Foundation Trust, Oxford, UK

[4] Peking University Health Science Centre, School of Public Health, Beijing, China

[5] NIHR Oxford Health Biomedical Research Centre, Warneford Hospital, Oxford, UK

[6] Discipline of Public Health and Primary Care, Institute of Population Health, School of Medicine, Trinity College Dublin, the University of Dublin, Dublin, Ireland

* **MG** and **PCD** are guarantors of the study.

Corresponding author:

Min Gao, PhD

Email: min.gao@phc.ox.ac.uk

University of Oxford, Nuffield Department of Primary Care Health Sciences, Radcliffe Observatory Quarter, Oxford, UK

Supplementary material 1

Updated search report

We searched Medline(OvidSP)[1946-present], Embase(OvidSP)[1974-present] and Science Citation Index(Web of Science Core Collection)[1900-present] from database inception to 4th May 2022, with a complete update run on **8th January 2024** and **8th January 2024** . The search strategy combines free-text keywords and subject headings where available for our main concepts: adiposity indices/cardiovascular disease, mental health disorders and mendelian randomisation, full strategies available in Appendix. No limits were placed on the search. Results were exported to Covidence for de-duplication of results and screening.

Table 1 Updated search results in Medline (Ovid MEDLINE® Epub Ahead of Print, In-Process & Other Non-Indexed Citations, Ovid MEDLINE® Daily and Ovid MEDLINE®) 1946 to present

Order	Terms	Number of studies
1	exp Overweight/	299310
2	exp body fat distribution/ or body mass index/ or body weight/ or sagittal abdominal diameter/ or exp waist circumference/ or waist-height ratio/ or skinfold thickness/ or waist-hip ratio/	367014
3	exp Adipose Tissue/	118675
4	(obes* or overweight or adipos*).ti,ab,kf.	563113
5	(body weight or body fat or fat mass or body composition or body mass index or bmi or "waist hip ratio" or "waist to hip ratio" or waist circumference).ti,ab,kf.	680965
6	1 or 2 or 3 or 4 or 5	1240340
7	Cardiovascular Diseases/	195616
8	heart diseases/ or exp myocardial ischemia/	561887
9	vascular diseases/ or cerebrovascular disorders/ or exp stroke/	278496
10	exp Diabetes Mellitus, Type 2/	192871
11	Hypertension/	270644
12	exp Hyperlipidemias/	73040
13	exp Lipoproteins/	169441
14	((cardiovascular or heart or card* or coronary or myocardi* or isch?mi* or vascular or cerebrovascular) adj2 disease?).ti,ab,kf.	711054
15	((myocardi* adj2 (infarct* or isch?emi*)) or angina or stroke).ti,ab,kf.	642538
16	diabet*.ti. or ((type 2 or type ii or noninsulin dependent or non-insulin dependent or adult onset or matur* onset) adj2 diabet*).ti,ab,kf.	544728
17	(hypertens* or high blood pressure).ti,ab,kf.	580668
18	(hyperlipid* or hypercholesterol* or cholesterol? or lipid? or triglyceride? or apolipoprotein?).ti,ab,kf.	952324

19	7 or 8 or 9 or 10 or 11 or 12 or 13 or 14 or 15 or 16 or 17 or 18	3265858
20	6 or 19	4107681
21	mental disorders/ or exp anxiety disorders/ or exp "bipolar and related disorders"/ or exp "feeding and eating disorders"/ or exp mood disorders/ or exp "schizophrenia spectrum and other psychotic disorders"/	600978
22	Mentally Ill Persons/	6486
23	((mental* or psychiatr*) adj2 (disease? or disorder? or health or ill*)).ti,ab,kf.	440371
24	(depress* or mood disorder? or anxiety or phobia? or phobic or obsessive compulsive or panic or bipolar or schizophren* or psychos?s).ti,ab,kf.	1037893
25	21 or 22 or 23 or 24	1485599
26	20 and 25	156487
27	Mendelian Randomization Analysis/	11163
28	((mendel* adj3 random*) or (instrumental adj3 variable?)).ti,ab,kf.	22747
29	27 or 28	22984
30	26 and 29	551

Table 2 Search results in Embase 1974 to present

Order	Search terms	Number of studies
1	exp obesity/	789224
2	body composition/ or abdominal circumference/ or adipose tissue thickness/ or body adiposity index/ or body fat percentage/ or body mass/ or body weight/ or fat free mass/ or fat mass/ or hip circumference/ or sagittal abdominal diameter/ or total body cholesterol/ or total body fat/ or waist circumference/ or waist hip ratio/ or waist to height ratio/ or weight height ratio/	1211130
3	exp Adipose Tissue/	240227
4	(obes* or overweight or adipos*).ti,ab,kf.	839870
5	(body weight or body fat or fat mass or body composition or body mass index or bmi or "waist hip ratio" or "waist to hip ratio" or waist circumference).ti,ab,kf.	1103304
6	1 or 2 or 3 or 4 or 5	2177116
7	Cardiovascular Disease/	433028
8	heart disease/ or exp ischemic heart disease/	983194
9	vascular disease/ or cerebrovascular disease/ or exp cerebrovascular accident/	648043
10	exp non insulin dependent diabetes mellitus/	431108

11	Hypertension/	890281
12	disorders of lipid and lipoprotein metabolism/ or exp hyperlipidemia/	230630
13	exp lipoprotein/	352893
14	((cardiovascular or heart or card* or coronary or myocardi* or isch?mi* or vascular or cerebrovascular) adj2 disease?).ti,ab,kf.	1054439
15	((myocardi* adj2 (infarct* or isch?emi*)) or angina or stroke).ti,ab,kf.	992847
16	diabet*.ti. or ((type 2 or type ii or noninsulin dependent or non-insulin dependent or adult onset or matur* onset) adj2 diabet*).ti,ab,kf.	783707
17	(hypertens* or high blood pressure).ti,ab,kf.	930349
18	(hyperlipid* or hypercholesterol* or cholesterol? or lipid? or triglyceride? or apolipoprotein?).ti,ab,kf.	1231276
19	7 or 8 or 9 or 10 or 11 or 12 or 13 or 14 or 15 or 16 or 17 or 18	4918875
20	6 or 19	6277043
21	mental disease/ or exp anxiety disorder/ or exp mood disorder/ or exp schizophrenia spectrum disorder/ or exp bipolar disorder/ or exp eating disorder/	1462856
22	mental patient/	31834
23	((mental* or psychiatr*) adj2 (disease? or disorder? or health or ill*)).ti,ab,kf.	559127
24	(depress* or mood disorder? or anxiety or phobia? or phobic or obsessive compulsive or panic or bipolar or schizophren* or psychos?s).ti,ab,kf.	1419205
25	21 or 22 or 23 or 24	2210680
26	20 and 25	345734
27	mendelian randomization analysis/ or instrumental variable analysis/	23740
28	((mendel* adj3 random*) or (instrumental adj3 variable?)).ti,ab,kf.	26378
29	27 or 28	28213
30	26 and 29	1104
31	limit 30 to "remove clinical trial (clinicaltrials.gov) records"	1098

Table 3 Search results in Science Citation Index 1900 to present

Order	Search terms	Number of studies
1	TS=(obes* OR overweight OR adipos*) OR TS=("body weight" or "body fat" or "fat mass" or "body composition" or "body mass index" or bmi or "waist hip ratio" or "waist to hip ratio" or "waist circumference")	1178027
2	TS=((cardiovascular or heart or card* or coronary or myocardi* or ischaemi* or ischemi* or vascular or cerebrovascular) NEAR/2 disease*) OR TS=((myocardi* NEAR/2 (infarct* or ischaemi* or ischemi*)) or angina or stroke) OR TI=diabet* OR TS=(("type 2" or "type ii" or "noninsulin dependent" or "non-insulin dependent" or "adult onset" or "maturity onset" or "mature onset") NEAR/2 diabet*) OR TS=(hypertens* or "high blood pressure") OR TS=(hyperlipid* or hypercholesterol* or cholesterol* or lipid or lipids or triglyceride* or apolipoprotein*)	3489761
3	#1 or #2	4272007

4	TS=((mental* or psychiatr*) NEAR/2 (disease* or disorder* or health or ill*)) OR TS=(depress* or "mood disorder*" or anxiety or phobia or phobias or phobic or obsessive compulsive or panic or bipolar or schizophren* or psychosis or psychoses)	1422515
5	TS=((mendel* NEAR/3 random*) or (instrumental NEAR/3 variable*))	27571
6	#3 AND #4 AND #5	631

Table 4 Updated search results of included databases

Database	Platform	Coverage	Hits: 04/05/2022	Hits: 08/01/2024	Hits: 22/09/2025	Final Hits
Embase	OvidSP	1974-present	315	589	1098	2002
Medline	OvidSP	1946-present	156	295	551	1002
Science Citation Index	Web of Science Core Collection	1900-present	237	343	631	1211
**Total:**			**708**	**1227**	**2280**	**4215**
Duplicates removed in Covidence:			305	902	1695	2902
**Final Total:**			**403**	**325**	**585**	**1313**

Supplementary material 2

Table 1 Quality assessment tool for Mendelian randomisation studies

**Section**	**Items**	**Criteria**			
		-	- +	+	Answer
**1.Measurement of exposure and outcome**	Type of measure for exposure	Unclear	Self-reported and measured	Measured	
	Type of measure for outcome	Single or a few (survey) question(s); self-reported (yes/no)	Validated, multi-item survey questionnaire; self-reported and Clinical diagnosis	Clinical diagnosis only	
**2.Selection of genetic variants**	p-value threshold	Unclear	≥5E-08	≤5E-08	
	F statistic sufficient	Not reported	<10	≥10	
	% variance explained	Unclear	Low or moderate (≤3%)	High (≥3%)	

**3.A. Analysis (One-sample MR)**				**3.B. Analysis (Two-sample MR)**			
	**-**	**+**	**Answer**		**-**	**+**	**Answer**
Bidirectional effects tested	Not tested	Tested		Bidirectional effects tested	Not tested	Tested	

Non-linearity assessed for continuous exposures	Not tested	Tested		Non-linearity assessed for continuous exposures	Not tested	Tested	
Tests for heterogeneity (e.g. Sargan test or Cochran's Q)	Not tested	Tested		Tests for heterogeneity (e.g. Sargan test or Cochran's Q)	Not tested	Tested	
Any attempt to do pleiotropy-robust sensitivity analysis	No	Yes		Any attempt to do pleiotropy-robust sensitivity analysis	No	Yes	
Results broadly similar amongst all MR analyses	No	Yes		Results broadly similar amongst all MR analyses	No	Yes	
				Mention of SNP harmonisation across samples	No/unclear	Yes	
				Estimates SNP-exposure and SNP-outcome are from comparable populations (e.g similar ancestry)	No/unclear	Yes	

Supplementary material 3

Table 1 Quality assessment results of included studies

Author	Part 1.1Type of measure for exposu	Part 1.2Type of measure for outcom	Part 1 (overall score)	Part 2.1p-value threshold	Part 2.2F statistic sufficient	Part 2.3 variance explained sufficient	Part 2 (overall score)	Part 3.A.1 Bidirectional effects teste	Part 3.A.2 Non-linearity assessed for continuous exposures	Part 3.A.3 Tests for heterogeneity (e.g. Sargan test or Cochran's Q)	Part 3.A.4 Any attempt to do pleiotropy-robust sensitivity analysis	Part 3.A.5 Results broadly similar amongst all MR analyse	Part 3.A (overall score)	Part 3.B.1 Bidirectional effects tested	Part 3.B.2 Non-linearity assessed for continuous exposures	Part 3.B.3 Tests for heterogeneity (e.g. Sargan test or Cochran's C	Part 3.B.4 Any attempt to do pleiotropy-robust sensitivity analysis	Part 3.B.5 Results broadly similar amongst all MR analyse	Part 3.B.6 Mention of SNP harmonisation across sampl	Part 3.B.7 Estimates SNP-exposure and SNP-outcome are from comparable populations (e.g similar ancestry)	Part 3.B (overall scor
Amin 2020	- +	- +	- +	+	+	- +	+	-	+	-	-	N/A	-	N/A	N/A	N/A	N/A	N/A	N/A	N/A	N/A
Aoki 2022	- +	+	+	+	-	-	-	N/A	N/A	N/A	N/A	N/A	N/A	-	-	+	+	+	-	+	+
Ardissino 2024	- +	+	+	+	+	+	+	N/A	N/A	N/A	N/A	N/A	N/A	-	-	+	+	+	+	+	+
Byg 2022	+	+	+	+	-	- +	- +	N/A	N/A	N/A	N/A	N/A	N/A	+	+	-	+	+	-	+	+
Cai 2024	- +	- +	- +	+	+	+	+	N/A	N/A	N/A	N/A	N/A	N/A	-	-	+	+	+	-	+	+
Carvalho 2021	- +	- +	- +	+	+	+	+	N/A	N/A	N/A	N/A	N/A	N/A	+	+	-	-	N/A	+	+	+
Casanova 2021	- +	-	- +	+	+	- +	+	-	+	-	-	+	-	-	+	-	+	+	+	+	+
Chen 2023	- +	+	- +	+	+	-	+	N/A	N/A	N/A	N/A	N/A	N/A	+	-	+	+	+	+	+	+
Chen 2025	- +	- +	- +	-	-	-	-	N/A	N/A	N/A	N/A	N/A	N/A	+	-	-	-	-	-	-	-
Day 2018	- +	- +	- +	+	+	+	+	N/A	N/A	N/A	N/A	N/A	N/A	-	+	-	+	+	+	+	+
Ding 2022	- +	+	+	+	+	-	+	N/A	N/A	N/A	N/A	N/A	N/A	+	+	+	+	+	-	+	+
Fan 2025	- +	-	- +	+	+	-	+	N/A	N/A	N/A	N/A	N/A	N/A	+	-	+	+	+	-	+	+
Guo 2024	+	+	+	+	-	-	-	N/A	N/A	N/A	N/A	N/A	N/A	-	-	+	+	+	+	+	+
Hartwig 2016	- +	+	+	+	+	+	+	N/A	N/A	N/A	N/A	N/A	N/A	-	+	-	+	+	+	+	+
He ML 2023	+	+	+	+	+	-	+	N/A	N/A	N/A	N/A	N/A	N/A	+	-	+	+	+	+	+	+
He RX 2023	- +	- +	- +	+	+	-	+	N/A	N/A	N/A	N/A	N/A	N/A	+	-	+	+	+	+	+	+
Huang 2024	- +	+	+	+	+	-	+	N/A	N/A	N/A	N/A	N/A	N/A	+	-	+	+	-	-	+	+
Hubel 2019	+	+	+	+	-	-	-	N/A	N/A	N/A	N/A	N/A	N/A	+	+	-	+	N/A	+	+	+
Hung 2014	- +	+	+	- +	+	- +	- +	-	+	-	-	N/A	-	N/A	N/A	N/A	N/A	N/A	N/A	N/A	N/A
Jokela 2012	+	- +	+	- +	+	- +	- +	-	+	-	-	N/A	-	N/A	N/A	N/A	N/A	N/A	N/A	N/A	N/A
Kappelmann 2021	- +	- +	- +	+	+	+	+	N/A	N/A	N/A	N/A	N/A	N/A	-	-	+	+	+	+	+	+
Karageorgiou 2023	+	- +	+	+	+	+	+	+	+	+	+	+	+	N/A	N/A	N/A	N/A	N/A	N/A	N/A	N/A
Khandaker 2020	+	-	- +	- +	+	+	+	-	-	-	-	+	-	N/A	N/A	N/A	N/A	N/A	N/A	N/A	N/A
Khani 2023	- +	+	+	+	-	-	-	N/A	N/A	N/A	N/A	N/A	N/A	+	-	+	+	+	+	+	+
Kivimaki 2011	+	- +	+	-	-	-	-	N/A	N/A	N/A	N/A	N/A	N/A	-	+	-	-	N/A	-	+	-
Lawlor 2011	+	-	- +	-	+	-	-	N/A	N/A	N/A	N/A	N/A	N/A	-	+	-	-	N/A	-	+	-
Luo 2024	- +	- +	- +	+	+	+	+	N/A	N/A	N/A	N/A	N/A	N/A	-	-	+	+	+	-	+	+
Ma 2025	- +	+	+	+	+	+	+	N/A	N/A	N/A	N/A	N/A	N/A	+	-	+	+	+	-	+	+
Martin 2022	- +	+	+	+	+	+	+	N/A	N/A	N/A	N/A	N/A	N/A	-	+	+	+	+	+	+	+
Meng 2024	- +	- +	- +	+	-	-	-	N/A	N/A	N/A	N/A	N/A	N/A	+	+	+	+	+	-	+	+
O'Loughlin 2023	- +	- +	- +	+	+	+	+	-	+	+	+	+	+	-	+	+	+	+	+	+	+
Palmos 2023	- +	- +	- +	+	+	-	+	N/A	N/A	N/A	N/A	N/A	N/A	+	-	+	+	+	-	+	+
Pathak 2025	+	- +	+	+	+	+	+	N/A	N/A	N/A	N/A	N/A	N/A	+	-	+	+	+	+	+	+
Perry 2021	- +	+	+	+	+	+	+	N/A	N/A	N/A	N/A	N/A	N/A	+	+	+	+	+	+	+	+
Peters 2020	- +	+	+	- +	+	- +	- +	N/A	N/A	N/A	N/A	N/A	N/A	+	-	+	+	+	+	+	+
Pistis 2021	- +	+	+	+	+	+	+	N/A	N/A	N/A	N/A	N/A	N/A	-	-	-	+	+	+	+	+
Polimanti 2017	- +	+	+	+	+	-	+	N/A	N/A	N/A	N/A	N/A	N/A	+	-	+	+	+	-	+	+
Qi 2019	- +	- +	- +	+	-	-	-	N/A	N/A	N/A	N/A	N/A	N/A	+	+	-	+	+	+	+	+
Qin 2025	+	+	+	+	+	+	+	N/A	N/A	N/A	N/A	N/A	N/A	-	-	+	+	-	-	+	-
Rees 2019	- +	+	+	+	+	+	+	N/A	N/A	N/A	N/A	N/A	N/A	+	+	-	+	+	+	+	+
Richardson 2019	+	+	+	+	-	-	-	N/A	N/A	N/A	N/A	N/A	N/A	+	+	+	+	N/A	+	+	+
So 2019	- +	+	+	+	+	- +	+	N/A	N/A	N/A	N/A	N/A	N/A	+	+	+	+	+	+	+	+
Speed 2019	+	- +	+	+	-	+	+	N/A	N/A	N/A	N/A	N/A	N/A	+	+	-	+	N/A	+	+	+
Tyrrell 2019	+	- +	+	+	+	- +	+	-	+	-	-	+	-	-	+	-	+	+	-	+	+
vandenBroek 2018	- +	+	+	+	-	- +	- +	N/A	N/A	N/A	N/A	N/A	N/A	+	+	+	+	+	+	+	+
Walter 2015a	- +	-	- +	-	-	- +	-	N/A	N/A	N/A	N/A	N/A	N/A	-	+	-	-	N/A	-	+	-
Walter 2015b	- +	- +	- +	-	-	- +	-	N/A	N/A	N/A	N/A	N/A	N/A	-	+	-	-	N/A	-	+	-
Willage 2018	- +	- +	- +	+	-	-	-	-	+	-	-	N/A	-	N/A	N/A	N/A	N/A	N/A	N/A	N/A	N/A
Wray 2018	- +	- +	- +	+	-	-	-	N/A	N/A	N/A	N/A	N/A	N/A	+	-	+	+	+	-	+	+
Xiao 2024	- +	+	+	+	+	+	+	N/A	N/A	N/A	N/A	N/A	N/A	+	+	+	+	+	+	+	+
Yang 2025	+	+	+	+	+	+	+	N/A	N/A	N/A	N/A	N/A	N/A	+	-	+	+	+	+	+	+
Yu 2023	+	+	+	+	+	-	+	N/A	N/A	N/A	N/A	N/A	N/A	+	-	+	+	+	-	+	+
Yuan 2025	- +	+	+	+	+	-	+	N/A	N/A	N/A	N/A	N/A	N/A	+	-	+	+	+	-	+	+
Zhan 2025	+	- +	+	+	+	-	+	N/A	N/A	N/A	N/A	N/A	N/A	+	-	+	+	+	-	+	+
Zhang 2021	- +	- +	- +	+	+	- +	+	N/A	N/A	N/A	N/A	N/A	N/A	+	-	-	+	+	+	+	+
Zhang 2024	- +	+	+	+	+	-	+	N/A	N/A	N/A	N/A	N/A	N/A	+	-	+	+	+	-	+	+
Zhou 2025	- +	- +	- +	+	+	+	+	N/A	N/A	N/A	N/A	N/A	N/A	+	-	+	+	+	+	+	+

Supplementary material 4

Table 1 Study cohorts overlap and sample size order	
**Variables**	**Database source**
BMI GWAS	GIANT (Pulit. et al., 2019) > GIANT (Turcot. et al., 2018) > GWAS(Yengo. et al., 2018) > GIANT (Shungin. et al., 2015) > GIANT (Locke. et al., 2014)
Depression GWAS	Psychiatric Genomics Consortium (Als et al., 2023) > Psychiatric Genomics Consortium (Howard, et al., 2019) > Psychiatric Genomics Consortium (Howard, 2019) without 23andMe > Psychiatric Genomics Consortium (Wray et al., 2018) > Psychiatric Genomics Consortium (Ripke,2013)
Bipolar disorder GWAS	Psychiatric Genomics Consortium (O'Connell et al., 2025) > Psychiatric Genomics Consortium (Mullins, 2021)> Psychiatric Genomics Consortium (Stahl et al., 2019)> GWAS (Hou et al., 2016) > Psychiatric Genomics Consortium (Sklar,2011)
Obsessive compulsive disorder	Psychiatric Genomics Consortium (Arnold, 2018) > GWAS(Mattheisen,2015)
PTSD	Psychiatric Genomics Consortium and Million Veterans Programme metan-analysis for PTSD (PGC-MVP)(Stein et al., 2021) > Psychiatric Genomics Consortium for PTSD freeze 1.5 dataset (PGC-PTSD) (Nievergelt et al., 2019)
Schizophrenia	Psychiatric Genomics Consortium (Trubetskoy, 2022) > Psychiatric Genomic Consortium (Pardinas, 2018) > Psychiatric Genomics Consortium (Ripke,2014)
Note: the sample size order is determined by the population size of the cohort	
